# A Meta-Analysis of Relationships between Measures of Wisconsin Card Sorting and Intelligence

**DOI:** 10.3390/brainsci9120349

**Published:** 2019-11-29

**Authors:** Bruno Kopp, Natasha Maldonado, Jannik F. Scheffels, Merle Hendel, Florian Lange

**Affiliations:** 1 Department of Neurology, Hannover Medical School, Carl-Neuberg-Straße 1, 30625 Hannover, Germany; n.maldonado@posteo.de (N.M.); scheffels.jannik@mh-hannover.de (J.F.S.); merle_hendel@t-online.de (M.H.); florian.lange@kuleuven.be (F.L.); 2 Behavioral Engineering Research Group, KU Leuven, Naamsestraat 69, 3000 Leuven, Belgium

**Keywords:** Wisconsin Card Sorting Test, intelligence, executive function, shifting, meta-analysis, psychometrics, validity

## Abstract

The Wisconsin Card Sorting Test (WCST) represents a widely utilized neuropsychological assessment technique for executive function. This meta-analysis examined the discriminant validity of the WCST for the assessment of mental shifting, considered as an essential subcomponent of executive functioning, against traditional psychometric intelligence tests. A systematic search was conducted, resulting in 72 neuropsychological samples for the meta-analysis of relationships between WCST scores and a variety of intelligence quotient (IQ) domains. The study revealed low to medium-sized correlations with IQ domains across all WCST scores that could be investigated. Verbal/crystallized IQ and performance/fluid IQ were indistinguishably associated with WCST scores. To conclude, the WCST assesses cognitive functions that might be partially separable from common conceptualizations of intelligence. More vigorous initiatives to validate putative indicators of executive function against intelligence are required.

## 1. Introduction

Psychological functions of the frontal lobes of the human brain remain enigmatic despite decades of research (see [1,2,3,4,5] for overviews). A substantial part of this research was based on the Wisconsin Card Sorting Test (WCST), which was originally developed in the 1940s [6,7]. The WCST requires sorting cards and using feedback to shift between different task rules. It consists of cards depicting simple geometric figures that vary in color, shape, and number. Examinees have to sort cards in accordance with one of three viable rules, i.e., according to the color, shape, or number of the depicted object(s). In order to identify the currently valid sorting rule, examinees have to rely on verbal feedback, which is provided by the examiner on each trial. Positive feedback indicates that cards were matched according to the correct rule on the current trial, whereas negative feedback indicates that the applied rule was invalid. The examiner changes the task rule after a number of successively correct card sorts have been conducted by the examinee. In this regard, the WCST bears similarities to task-switching paradigms that are often utilized in experimental psychology ([8] for overview; [9,10,11]). Popular WCST scores include (1) the number of completed runs of correct card sorts (usually referred to as ‘categories’), (2) the number of perseverative errors or responses, (3) the number of non-perseverative errors, (4) the number of failures to maintain a rule (or ‘set’), and (5) the number of total errors (see [12,13] for overviews).

The WCST was introduced to neuropsychology in the 1950s for assessing higher visual functions [14]. Based on this study, it was originally thought that performance on the WCST was sensitive to (traumatic) posterior brain lesions. However, Milner’s seminal study [15] revealed that the presence of (massive, unilateral) prefrontal excisions was associated with frequently occurring rule perseverations on the WCST, despite preserved indicators of intelligence. (Milner’s publication [15] does not designate the specific intelligence test (e.g., FSIQ, VIQ, PIQ) that was utilized for the quantification of intelligence. Given that the patients were doubtlessly studied in the 1950s, and given the information provided in the related paper [16], the most probable interpretation of ‘the IQ’ in Milner’s study is that this acronym represents the IQ that was obtained from the Wechsler–Bellevue Intelligence Scale [17]. A longstanding criticism of intelligence tests such as the early versions of the Wechsler batteries (up to WAIS-R) was that there was disproportionate emphasis on measures of Gc. By contrast, these early versions of the Wechsler batteries had weaker representation of measures of Gf.) Hence, this study laid the ground for the proposition that frontal lobe functions can be assessed behaviorally by means of the WCST [18,19,20]. In the decades to follow, the idea that the WCST measures frontal lobe functions was successively replaced by the conception of the WCST as a test of executive function (EF), thereby relaxing the otherwise strong constraints about the neuroanatomical substrates of WCST performance (see [21,22,23,24,25] for reviews). EFs encompass higher cognitive functions, usually defined as a set of domain-general cognitive control mechanisms supporting goal-directed behavior (e.g., [26]), but their exact nature remains a matter of debate [27,28,29,30]. The unity/diversity model of EF, which represents a well-validated individual differences model of EF, proposes that specific EF factors of updating and shifting exist next to a general factor that is involved in all EF tasks [31,32,33,34]. Applying an early version of their model, Miyake and colleagues [35] showed that the number of perseverative errors committed on the WCST specifically reflected individual differences in the shifting factor.

Since the time of Milner’s study [15], the WCST has received several modifications (most notably by [36]) and multiple standardizations [37,38,39]. The availability of standardized test versions, as well as the prevalent acceptance of the EF construct, may have contributed to widespread dissemination of the WCST in clinical neuropsychology. The WCST is currently the most popular assessment instrument for EF [13]. Behavioral performance on the WCST is commonly interpreted in terms of mental shifting, a process which represents an important subcomponent of EF, and ensures cognitive flexibility in accordance with task requirements.

The dissociation between WCST performance and intelligence that has been reported in Milner’s study [15] might also have contributed to the widely held belief that EF represents a psychological construct that is separable from intelligence (e.g., [26]). While a detailed discussion of the facets of human intelligence goes beyond the scope of this article, a few remarks seem justified here. David Wechsler once defined intelligence as the “the global capacity of the individual to act purposefully, to think rationally and to deal effectively with his environment” [40] (p. 3). From this definition, it is evident that intelligence and EF share substantial conceptual overlap. 

Psychological science of the 20th century evidenced a controversy about the most reasonable theoretical model of intelligence [41]. Spearman initially identified a single general ability that he named *g* (for “general factor” [42,43], but see [44]). Meanwhile, a consensus regarding the dimensionality of intelligence has only been achieved insofar as most researchers agree with the assumption of a hierarchical structure of cognitive abilities that underlie intelligence, with *g* at its highest level. Cattell [45] distinguished two types of cognitive abilities that are relevant for intelligence in a revision of Spearman’s concept of *g*. Fluid intelligence (Gf) was hypothesized as the ability to solve novel problems by using reasoning, and crystallized intelligence (Gc) was hypothesized as a knowledge-based ability that was heavily dependent on education. After Horn [46] identified a number of broad cognitive abilities in a revision of the Gf-Gc theory, Carroll [47] proposed a hierarchical model with three levels, which is now known as the CHC (Cattell–Horn–Carroll) model of intelligence [48]. The bottom level consists of highly specialized, task-specific abilities. The second level consists of a number of broad cognitive abilities, including Gf and Gc. Carroll accepted Spearman’s concept of *g* as a representation of the highest level, affecting performance on any particular test solely via its influence on identified broad cognitive abilities [47]. The CHC model of intelligence forms the basis of many contemporary cognitive test batteries [49].

Regardless of one’s preferred theoretical model of intelligence, the most widely utilized tests of intelligence are the Wechsler Adult Intelligence Scale (WAIS) and the Wechsler Intelligence Scale for Children (WISC). The initial version of the WAIS was released in 1955 [50], followed by the WAIS-R (1981) [51], WAIS-III (1997) [52], and WAIS-IV (2008) [53]. The initial version of the WISC was released in 1949 [54], followed by the WISC-R (1974) [55], WISC-III (1991) [56], and WISC-IV (2003) [57]. Apart from an estimate of general intelligence (i.e., Full Scale IQ, FSIQ), Wechsler tests were often used to obtain sub-scores of Verbal intelligence (VIQ) and Non-verbal Performance intelligence (PIQ). The concepts of VIQ and PIQ are closely related to the CHC abilities Gc and Gf, respectively. (The verbal VIQ and the non-verbal PIQ represent concepts that are a little bit broader than Gc and Gf. In the case of the WAIS-R, the VIQ includes the subtests {information, comprehension, arithmetic, digit span, similarities, and vocabulary}, while the PIQ includes the subtests {picture arrangement, picture completion, block design, object assembly, and digit symbol}. This is similar for the WAIS-III because here the subtests that comprise the WAIS-III VIQ, which are labeled verbal comprehension {vocabulary, similarities, information, comprehension} and working memory {arithmetic, digit span, letter-number sequencing}, confer to Gc plus Gsm (short-term memory; see [58], Table 5). The subtests that comprise the WAIS-III PIQ, which are labeled perceptual organization {picture completion, block design, matrix reasoning} and processing speed {digit-symbol coding, symbol search}, confer to Gf, Gv (visuospatial abilities) plus Gs (processing speed; see [58], Table 5)).

Other IQ tests focus more directly on the assessment of CHC-compatible broad cognitive abilities. For example, the National Adult Reading Test [59] (NART) is often used to assess Gc in clinical neuropsychology, under the assumption that this education-dependent facet of intelligence is relatively insensitive to neurological alterations, and can thus serve as an estimate of the premorbid level of crystallized intelligence [26]. Crawford et al. [60] found that the NART predicted 72% of WAIS-VIQ variance, but only 33% of the WAIS-PIQ variance. Raven’s Progressive Matrices [61] (RPM) and the Culture Fair Test [62] (CFT) are often considered as quintessential measures of fluid intelligence (e.g., [43]). The RPM and the CFT are also closely related to the PIQ since both tests utilize non-verbal materials. In the remainder of this article, we have thus considered the NART as a proxy for the VIQ (both rather focusing on the assessment of Gc), and the RPM/CFT as proxies for the PIQ (all rather focusing on the assessment of Gf). 

A number of authors have tried to unify intelligence and neuropsychological assessment based on the CHC model [58,63,64,65,66,67,68,69,70,71]. For example, Jewsbury et al. [65] showed that popular neuropsychological EF tests were subsumable under CHC broad cognitive abilities based on factor analytic methods, although particular EF tests were related to distinct CHC constructs. Most importantly in the present context, WCST perseverative errors were found to be related to GvGf, i.e., visuospatial (Gv) and fluid (Gf) facets of intelligence in that study (see also [64]). The conclusion that WCST performance and fluid intelligence are highly correlated was also corroborated by a neuropsychological study: Roca et al. [72] showed that when patients suffering from frontal lobe lesions and controls were matched on the CFT, i.e., on a measure of Gf, the frequency of WCST total errors no longer differed between these groups. The authors took these data to suggest that the unique variance in WCST performance was negligible once the variance that this measure shared with fluid intelligence was accounted for. The conclusion drawn by Roca and colleagues [72] lies in obvious conflict with Milner’s [15] assertion that the WCST allows for the detection of frontal dysfunctions in the absence of noticeable declines in intelligence.

The question to what degree WCST performance is separable from measures of intelligence is of vital importance for the concept of EF. EF would be an unnecessary psychological construct if discriminant validation of WCST scores against indicators of intelligence should fail. Cronbach characterized the issue in the following words: 

“To defend the proposition that a test measures a certain variable defined by a theory, one looks basically for two things. The first is *convergence* of indicators. […] The second kind of evidence is *divergence* of indicators that are supposed to represent different constructs. If a test is said to measure “ability to reason with numbers,” it should not rank pupils in the order a test of sheer computation gives, because the computation test cannot reasonably be interpreted as a reasoning test. The test interpretation should also be challenged if the correlation with a test of verbal reasoning is very high, because this would suggest that general reasoning ability accounts for the ranking, so that specialized ability to reason with numbers is an unnecessary concept.” ([73], p. 144; italics in the original text)

According to Cronbach’s example, the construct of numerical reasoning would be unnecessary in the case that discriminant validation against computational abilities or verbal reasoning should fail. In general, any worthwhile cognitive construct (e.g., numerical reasoning) requires discriminant validation against related cognitive constructs (e.g., computational ability, general reasoning). We referred to this prerequisite of designing an evidence-based cognitive architecture as ‘Cronbach’s hurdle’. Of importance for the present study, putative EF tests (such as the WCST) had to demonstrate discriminant validity against measures of intelligence in order for the EF construct to take the hurdle. The provision of empirical support for discriminant validity of the WCST has been a relatively neglected topic [74]. Some of the few exceptions to that rule were discussed above in detail. These studies had their methodological grounding in factor analytic methods [64,65,68,69,70], in regression methods [71], or in neuropsychological patient studies [15,72].

The present meta-analysis complements the hitherto available evidence with regard to the discriminant validity of WCST scores against intelligence. For that purpose, our meta-analysis focused on the correlations between popular WCST scores (i.e., number of categories, frequency of various types of errors) and a variety of IQ domains (i.e., FSIQ, PIQ, VIQ). Individual studies often fail to obtain reliable estimates of correlations due to insufficient sample sizes [75]. By pooling data from these studies using meta-analytical techniques, one cannot only arrive at more reliable correlation estimates, but also examines potential origins of between-study variability in the strength of these correlations [76]. Thus, the present meta-analysis of correlations between WCST scores and IQ domains informed the ongoing discussion (a) about the construct validity of the WCST, and, by way of this, (b) about the overlap between EF and intelligence in a more general sense.

## 2. Materials and Methods

### 2.1. Search Strategy

**Figure 1:**
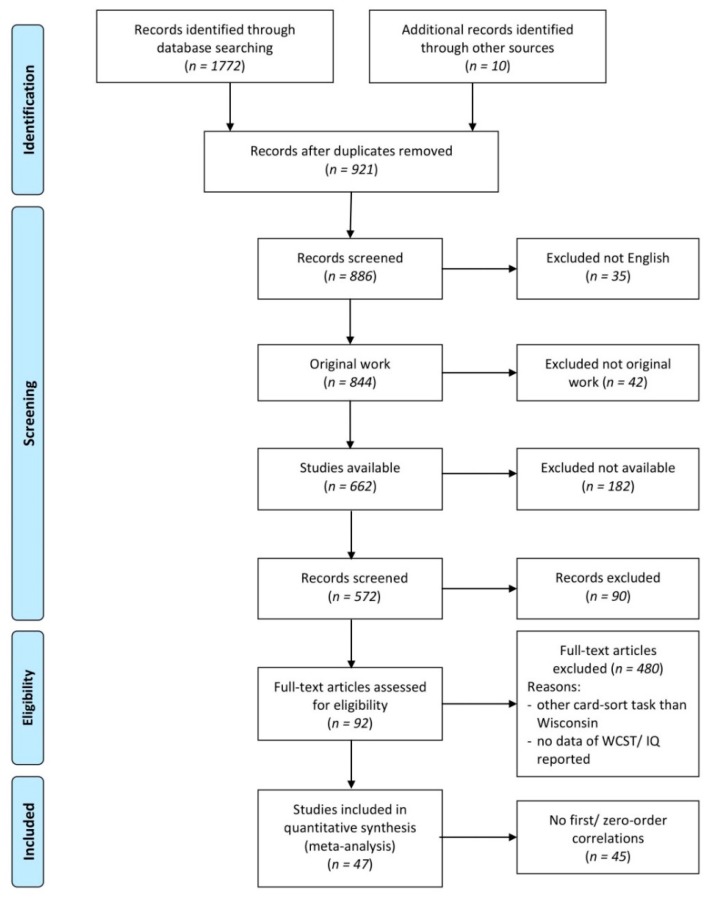
Flow chart depicting the selection of articles for our meta-analysis.

A systematic literature review was conducted in 2017 by MH and updated in July 2018 by NM. We searched for records including the term “card sort *” in combination with any of the following keywords regarding intelligence domains and tests: “intelligence”, “iq”, “fsiq”, “viq”, “piq”, “WAIS”, “WISC”, “normative”, “progressive matrices”, “Raven’s matrices” and “Raven’s”. PubMed (705 studies), Science Direct (326 studies), Web of Science (741 studies) and, in addition, the Compendium of Neuropsychological Tests [77] (10 studies) yielded a total of (1782 studies) hits for these combinations of search keywords (Figure 1). First of all, double appearances (861 studies) were excluded. Thirty-five additional papers were published in languages other than English and therefore had to be excluded. We screened the titles and abstracts of the remaining records and excluded studies that did not involve an assessment of original data from the WCST and intelligence domains (e.g., reviews or meta-analyses). 182 studies of the left over 844 studies remained inaccessible via local university libraries or open access. 

In total, we accessed 662 full texts, and we checked whether the data reported in these papers included correlation coefficients for the relationship between any scores of WCST performance and any domains of intelligence. At this step, studies were excluded when it became apparent that they did not administer the WCST, or when they did not report data from the WCST and at least one domain of intelligence. Papers that only reported test statistics for group difference involving WCST scores and intelligence domains, without reports of correlative relationships between WCST and intelligence, were also excluded at this step.

Of the remaining 92 studies, 45 did not report correlations between WCST scores and intelligence domains and were therefore excluded. The studies that had to be excluded at this final step either reported the results of multivariate statistics (e.g., regression analyses or factor analyses) that did not allow for the estimation of bivariate correlations, or they did not include a measure that could be utilized for estimating the FSIQ, VIQ, or PIQ domains. Forty-seven studies remained for the final meta-analysis.

### 2.2. Data Extraction and Coding

#### 2.2.1. WCST Scores

The extracted studies reporting correlations between WCST performance and intelligence reported a large variety of different WCST scores. To guarantee adequate statistical power for all analyses, we decided to focus on the WCST scores that had been reported in at least five independent studies. All those excluded (such as conceptual level responses or numbers of trials required to complete the first category) were found to be reported in a maximum of two studies. Analyzed scores included:

1. the number of categories completed (correct sequences of 6 or 10 consecutive correct matches to the criterion sorting category; the sequence length depends on the test version)
2. the frequency of perseverative errors or responses (persisting to respond to an incorrect stimulus characteristic)
3. the frequency of non-perseverative errors (errors that are not considered as perseverative errors)
4. the frequency of failures to maintain the set (e.g., when five or more consecutive correct matches are made, followed by at least one error prior to successfully completing the category) and
5. the frequency of total errors.

We did not distinguish between absolute and relative scores of the different error types (e.g., between the number and the percentage of perseverative errors). These scores are typically highly correlated and pooling data across these two types of measures allowed for a more powerful analysis of the relationship between the respective facet of WCST performance and intelligence. When a study reported both absolute and relative figures for a particular error type, we extracted the correlations involving the absolute figures. Similarly, to avoid redundancy and increase statistical power, we selected only one perseveration score for each study and did not further distinguish between perseverative errors and perseverative responses (see [21]). When multiple scores of perseveration were reported, we extracted the correlations involving perseverative errors [11]. One included study distinguished between two types of perseverative errors and we averaged correlation coefficients across both types to extract a single score representing perseveration for this study. Some studies did not report the total frequency of errors, but the total frequency of correct responses. For those studies, we changed the sign of the correlation coefficients involving the total frequency of correct responses to obtain an estimate for the correlation coefficients involving the total frequency of errors.

#### 2.2.2. IQ Domains

We distinguished between three domains of intelligence, verbal intelligence (VIQ), performance intelligence (PIQ), and full-scale intelligence (FSIQ). With regard to VIQ, most studies reported correlations involving VIQ scores from a version of the WAIS or WISC. When studies reported only correlations involving VIQ subdomains, this information was used to estimate the correlation between VIQ and WCST scores. When multiple VIQ subdomains were reported (e.g., similarities and information), we computed average correlations across them. When only a single VIQ subdomain was reported (e.g., vocabulary) we took its correlation as the best estimate of the correlation between VIQ and WCST performance. Vocabulary tests that are used to assess premorbid intelligence (i.e., the NART and the MWT [78]) were also considered to be measures of VIQ as was the Ammons Quick Test [79].

Similarly, values for the PIQ category were obtained by extracting reported Wechsler PIQ aggregate scores or by estimating PIQ based on the reported Wechsler PIQ subdomains. The remaining measures in the PIQ category included the CFT (and its matrix subtest), the RPM, the Shipley Abstraction test [80], the matrices subtest of the Stanford–Binet Intelligence Scale [81], and the reasoning subtest of Thurstone’s Primary Mental Abilities [82].

One study reported two indices of VIQ and two other studies reported two indices of PIQ. For these studies, we extracted the correlations involving the more common domain (i.e., the one that was more frequently reported in the other included studies) and conducted robustness tests using the alternative domain.

The FSIQ category consisted exclusively of FSIQ aggregate scores obtained from the intelligence tests of Wechsler and Kaufman [83]. Some studies reported data from established short versions of a Wechsler test and others created ad hoc short versions by combining scores from subtests of the VIQ and PIQ domains (e.g., Vocabulary and Block Design). They were all considered as FSIQ domains for the present set of analyses.

### 2.3. Correlation Coefficients

Given the selection of five WCST scores and three domains of intelligence, we extracted a theoretical maximum of 15 correlation coefficients per independent study sample. Some studies reported multiple correlation coefficients per measure combination as a result of investigating this correlation in independent subgroups (e.g., patients vs. control participants). For these studies, we extracted correlation coefficients separately for every independent sample of participants. Most of the included studies reported Pearson’s *r* or Spearman’s *rho* correlation coefficients, one study reported Kendall’s *tau*, and another study reported a mix of parametric and non-parametric correlations (see Table 1). 

We did not invert the sign of the extracted correlation coefficients, that is, positive correlations between IQ and the number of completed WCST categories and negative correlations between IQ and WCST error scores indicated that WCST performance improved with increasing IQ. When a study did not report the size of a correlation coefficient, but only that this coefficient was not statistically significant, we excluded this coefficient from our analyses. However, we ran additional robustness analyses to test whether our results changed when these coefficients were included as correlations of *r* = 0. 

### 2.4. Basic Meta-Analysis

Mean effect sizes and confidence intervals for the relationships between the five selected WCST scores and three selected intelligence domains were calculated using the random-effects model method (with inverse variance weights) proposed by Hedges and Vevea [130] and implemented by Field and Gillett [76]. Heterogeneity of effect sizes were examined using Cochran’s *Q* and the *I*^2^ index [131]. By comparing Cochran’s *Q* (estimated under fixed-effect assumptions) to a χ^2^ distribution, we tested whether heterogeneity among studies was significant. The *I*^2^ index served as an estimate of between-study heterogeneity in true effect sizes, with *I*^2^ values of about 25%, 50% and 75% indicating low, moderate, and high heterogeneity, respectively [131].

### 2.5. Moderator Analyses

We examined whether the strength of the correlation between domains of intelligence and WCST performance were moderated by the sample and study characteristics that were routinely reported in neuropsychological studies on the WCST-IQ relationship. To guarantee a minimum statistical power for these analyses, we focused on the correlations involving the two most frequently reported WCST scores, that is, the number of completed WCST categories and the frequency of perseverations. We extracted the following variables as potential moderators of the WCST-IQ relationship: (1) the mean age of participants, (2) the standard deviation of participants’ age, (3) the proportion of female participants in the sample, (4) clinical status, (5) the WCST version used, (6) the WCST administration method used, and (7) the intelligence test used.

Demographic variables (mean age, standard deviation of age as well as proportion of female participants) were treated as continuous predictors. For illustrative purposes, we also created three groups of studies including participants from different age categories (mean age <18 years, 18–50 years, >50 years) and repeated our analyses with mean age as a categorical predictor. Some studies reported WCST and IQ data from a sample that was smaller than the sample for which they provided demographic data (i.e., not all participants completed all neuropsychological measures). For these studies, we estimated the age and sex distribution of the sample of interest (i.e., the sample underlying the computation of WCST-IQ correlations) by extracting the demographic data for the total sample. When studies did not provide the standard deviation of participants’ age, standard deviations were estimated from range data (minimum, maximum) according to the procedure described by Wan, Wang, Liu, and Tong [132].

Regarding the moderating role of clinical status, we tested whether correlations between IQ domains and WCST scores were stronger in samples of patients with psychiatric or neurological disorders than they were in samples of healthy participants. Some studies reported correlation coefficients from mixed samples of patients and healthy controls. These studies were excluded for the analysis of the moderating role of clinical status.

With regard to the WCST version used in the individual studies, we distinguished (a) between the Heaton version [37,38] and the Nelson version [36] of the test and (b) between computerized and non-computerized WCST versions. IQ domains were contrasted based on their comprehensiveness. With regard to FSIQ, we distinguished between studies that used an abbreviated version (established or *ad hoc*) of an FSIQ test (i.e., an aggregate of a subset of subtests) and studies that used full version FSIQ tests. With regard to VIQ tests, we distinguished between pure vocabulary tests (NART, MWT-B, Ammons Quick Test, Wechsler vocabulary subtests) and more comprehensive VIQ tests (i.e., aggregates of at least two Wechsler subtests). With regard to PIQ tests, we distinguished between culture-reduced (matrices) tests (Cattell’s matrices, CFT, RPM, RCPM) and Wechsler scores (aggregated across at least two subtests).

The relationship between potential moderators and the size of the WCST-IQ correlation was examined using separate weighted multiple regression analyses (random-effects model with inverse variance weights [76]). Continuous moderator variables (i.e., mean age, standard deviation of age, proportion of female participants) were z-transformed to facilitate comparisons between regression coefficients.

### 2.6. Publication Bias Analysis

The Begg and Mazumdar rank correlation test was calculated as implemented in the syntax by Field and Gillett [76] to examine the relationship between effect sizes and their standard errors. A positive correlation between these two variables was indicative of a small-study effect (i.e., the tendency for studies with smaller samples to produce larger effect size estimates). Such an overrepresentation of small studies with large effect sizes can be the result of publication bias [133] and it would likely contribute to an overestimation of the true effect size. Note that this logic only applied when the effect size in question was positive (e.g., as expected for the correlation between WCST categories and IQ tests). With regard to negative average effect sizes (e.g., as expected for the correlation between WCST error scores and IQ domains), negative correlations between effect sizes and standard errors were indicative of small-study effects.

### 2.7. Partial Correlations

Partial correlations were used to examine a) the age-corrected relationship between WCST performance and IQ and b) the IQ-corrected relationship between age and WCST performance. Partial correlations were either directly extracted from the publication or calculated based on zero-order correlations using http://vassarstats.net/ [134]. Only 11 studies reported the information necessary to be included into one of our meta-analyses on partial correlations and we decided to pool these data across WCST scores and IQ domains. For this purpose, the former was recoded so that larger values indicated better performance. When partial correlations involving multiple WCST scores were reported, the average partial correlation across them was extracted.

## 3. Results

The results of our random-effects meta-analysis of the correlation between IQ domains and WCST performance is displayed in Table 2. An inspection of Table 2 reveals that most of the analyzed WCST scores were significantly related to all IQ domains. With the notable exception of weak correlations between IQ and WCST failures to maintain set, the size of WCST-IQ correlations ranged from small-to-medium (*r* = 0.19) to medium-to-large (*r* = 0.44) [135]. Correlations appeared to be stronger when they involved (a) a general (i.e., categories, total errors) versus more specific (i.e., perseverations, non-perseverative errors) WCST performance score, and (b) a general (i.e., FSIQ) versus more specific (i.e., VIQ, PIQ) domain of IQ. Note, however, that most of the 95% confidence intervals surrounding the corresponding effect sizes overlapped substantially.

Robustness analyses revealed that average correlation coefficients increased slightly (by *r* = 0.002 to *r* = 0.021) when alternative IQ tests were included (see Section 2.2.2) and decreased slightly (by *r* = 0.009 to *r* = 0.023) when coefficients that were described as non-significant were included as coefficients of *r* = 0. The negligible magnitude of these changes suggested that the results displayed in Table 2 were largely invariant to the analytical choices we made when extracting effect sizes from individual studies.

**Figure 2:**
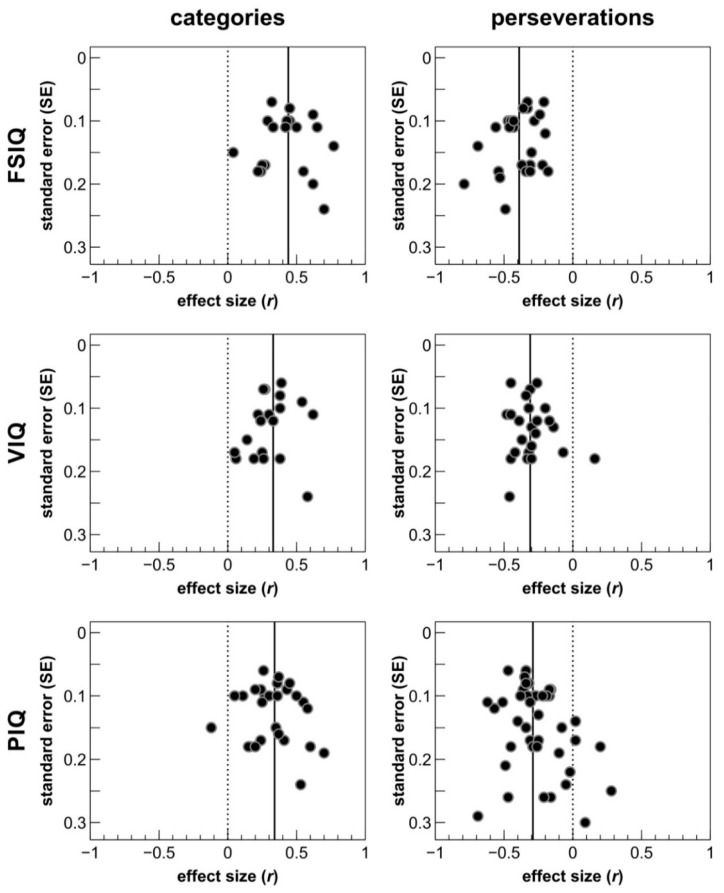
Funnel plots illustrating the relationships between effect sizes and their standard errors. Straight vertical lines indicate the average meta-analytical effect size obtained for the respective correlation between a WCST score (categories, perseverations) and intelligence domain (FSIQ, VIQ, PIQ). Dotted vertical lines display zero effects for comparison. WCST = Wisconsin Card Sorting Test, FSIQ = full scale IQ, VIQ = verbal IQ, PIQ = performance IQ.

Rank correlation tests did not find any of the significant WCST-IQ correlations to be significantly affected by small-study effects. In combination with the funnel plots displayed in Figure 2, these results suggest that meta-analytical correlation coefficients are unlikely to be substantially overestimated due to publication bias. This notion receives further support from the observation that many of the publications included in this meta-analysis reported non-significant correlations between WCST scores and IQ domains (see Table 2).

### 3.1. Moderator Analyses

Effect-size heterogeneity was moderate (i.e., around *I*² = 50%) for most of the analyzed WCST-IQ correlations. These results indicated that the size of these correlations may vary as a function of sample or study characteristics. Our moderator analyses (see Table 3) identified some of the factors that contributed to heterogeneity in the size of WCST-IQ correlations. 

**Figure 3:**
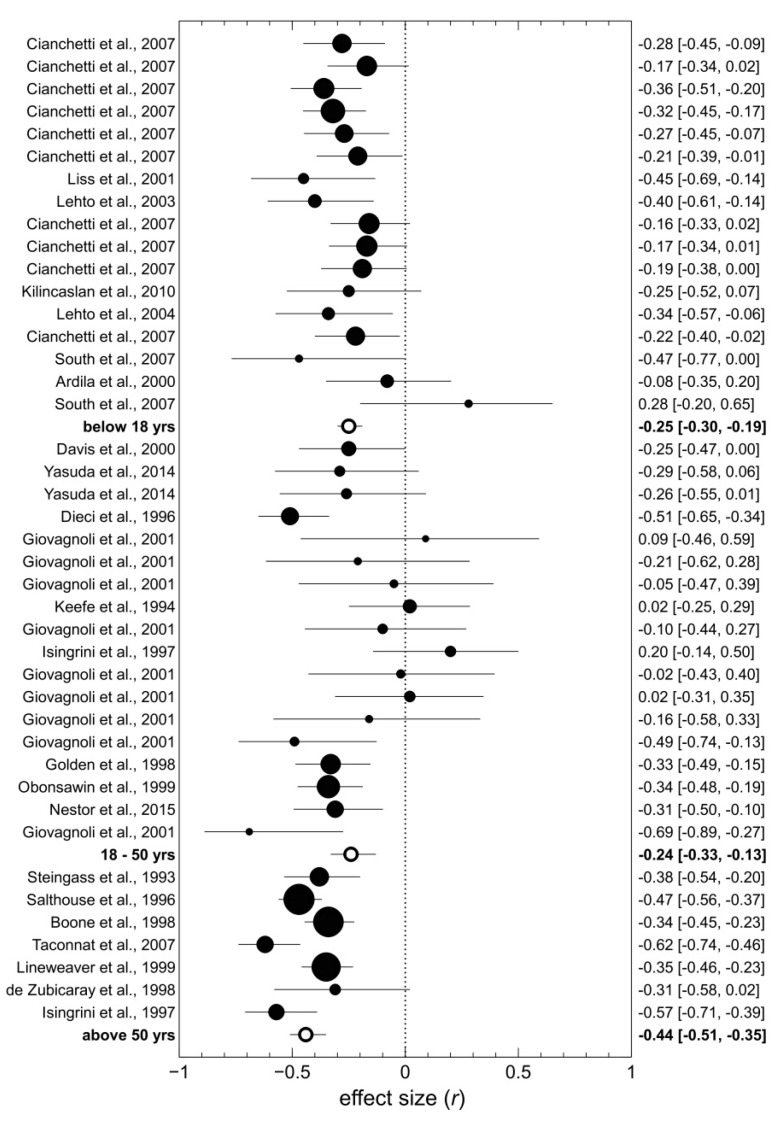
Effect sizes of the correlation between WCST perseverations and performance IQ as a function of participants’ mean age. Effect sizes are sorted from youngest (top) to oldest (bottom) sample. The size of black-filled circles is proportional to sample size. White-filled circles indicate average effect sizes across age groups. WCST = Wisconsin Card Sorting Test.

First, we found the correlation between WCST perseverations and PIQ domains to depend on the mean age of participants in the sample, β = −0.07, *t* (39) = −3.05, *p* = 0.004. Correlations were markedly stronger in samples with a mean age above 50 years, *k* = 7, *r* = −0.44, 95% CI [−0.51, −0.35], *I*^2^ = 57%, than they were in samples of younger adults (18−50 years), *k* = 18, *r* = −0.24, 95% CI [−0.33, −0.13], *I*^2^ = 49%, or in samples of children and adolescents (below 18 years), *k* = 17, *r* = −0.25, 95% CI [−0.30, −0.19], *I*^2^ = 4%, as shown in Figure 3.

Second, moderator analyses revealed the size of the correlation between WCST perseverations and FSIQ domains to be related to participants’ clinical status, *χ^2^*(1) = 4.76, *p* = 0.029. Correlations were stronger in samples of patients, *k* = 9, *r* = −0.49, 95% CI [−0.60, −0.36], *I*^2^ = 62%, than they were in samples of healthy individuals, *k* = 11, *r* = −0.34, 95% CI [−0.39, −0.27], *I*^2^ = 17%. 

Finally, we did not find the size of WCST-IQ correlations to depend significantly on the WCST version or IQ test administered in the original studies. This lack of effect-size difference cannot be considered conclusive given the small number of studies involved in these comparisons. However, it is worth noting that irrespective of the administered test versions, WCST-IQ correlations remained significant. For both categories and perseverations, we found exclusively significant correlations with all IQ domains for both Heaton versions, |*r*| = 0.33–0.46, all *p* < 0.001, and Nelson versions, |*r*| = 0.26–0.45, all *p* < 0.001, of the WCST. Similarly, all WCST-IQ correlations were substantial when involving traditional, non-computerized WCST variants, |*r*| = 0.29–0.47, all *p* < 0.001, as well as in the small number of studies involving computerized WCST versions, |*r*| = 0.23–0.34, all *p* < 0.026. With regard to IQ test variants, comprehensive Wechsler-type measures of FSIQ, VIQ, and PIQ were found to correlate significantly with WCST categories and perseverations, |*r*| = 0.30–0.43, all *p* < 0.001. Correlations were also substantial (*p* < 0.001) in the smaller samples of studies administering short-version FSIQ tests, categories: *r* = 0.37; perseverations: *r* = −0.31, vocabulary VIQ tests as indicators of premorbid intelligence, categories: *r* = 0.27; perseverations: *r* = −0.29, or culture-reduced (matrices) PIQ tests, categories: *r* = 0.32, 95%, perseverations: *r* = −0.27.

### 3.2. Partial Correlation Analyses

Meta-analysis of partial correlation coefficients revealed a significant relationship between WCST and IQ performance when controlling for age, *k* = 10, *r* = 0.36, 95% CI [0.25, 0.46], *I*^2^ = 70%. A rank correlation test did not find this relationship to be significantly affected by small-study effects, *r* = 0.20, *p* = 0.421. The correlation between age and WCST performance was not significant when controlling for IQ, *k* = 9, *r* = −0.04, 95% CI [−0.24, 0.17], *I*^2^ = 91%. Of note, this overall null-correlation resulted from a positive IQ-corrected relationship between age and WCST performance in samples of children and adolescents, *k* = 3, *r* = 0.35, 95% CI [0.15, 0.52], *I*^2^ = 67%, and a negative IQ-corrected relationship between age and WCST performance in adult samples, *k* = 6, *r* = −0.22, 95% CI [−0.32, −0.11], *I*^2^ = 49%.

## 4. Discussion

The present meta-analysis examined discriminant validity of WCST scores against common domains of intelligence. We found robust, low to medium-sized correlations between WCST performance and IQ across all WCST scores and IQ domains that we investigated. Solely the average correlations between WCST failures to maintain set and IQ amounted to coefficients very close to zero. Average correlations between WCST non-perseverative errors and IQ were higher (|*r|* = 0.19–0.30), and correlations between the most commonly utilized WCST scores (number of categories, total errors, perseverations) and IQ were generally the highest. Average correlations between these WCST scores and FSIQ were somewhat higher (|*r|* = 0.39–0.44) than those between them and VIQ (|*r|* = 0.31–0.37) and PIQ (|*r|* = 0.29–0.36), respectively. Taken together, the present meta-analysis revealed modest correlations between most of the WCST scores and IQ domains, based on sample sizes that varied between *N* = 260 and *N* = 3256. 

### 4.1. Discriminant Validity of the WCST

If one thinks about the observed correlations in terms of the proportion of the variance in WCST scores that is predictable from IQ, the calculation of *r squared* reveals about 0 (failures to maintain set) to 19 (categories) percent shared variance, leaving 81 to 100 percent unique WCST variance, i.e., variance unaccounted for by common measures of intelligence. Our findings therefore suggest that WCST and FSIQ, VIQ, PIQ represent partially separable measures of cognitive abilities. One possibility to account for these results lies in the referral to the unity/diversity model of EF that we shortly presented in the Introduction. According to the latest revision of the model, performance on EF tasks can be accounted for by a general EF factor, an updating-specific factor, and a shifting-specific factor. Both the general EF factor and the updating-specific factor seem to share substantial variance with measures of intelligence. In contrast, the shifting factor, which has been shown to underlie perseverative errors on the WCST [35], appears to be largely unrelated to intelligence [31,136].

A second possibility to account for these results is grounded in the distinction between measures and constructs, and the argument that less than perfect measurement reliability attenuates the actual correlations that may exist at the construct level [137,138]. Yet, even when we corrected for potential attenuation of the correlations that might result from imperfect reliabilities of WCST scores and IQ domains, the unique portion of WCST variance surpassed the portion of WCST variance that was shared with variance in IQ. As an example, consider the strongest correlation that we found in our meta-analysis, that is, the correlation between WCST categories and FSIQ (*r_xy_* = 0.44). Based on a quite conservative estimate for the reliability of the IQ tests that were studied (*r_xx_* = 0.90), and a computed average reliability of WCST categories (about *r_yy_* = 0.60; see Table A1 and Table A2 in the Appendix A), this observed correlation can be ‘disattenuated’ to yield an estimated correlation of *r_xtyt_* = 0.60 at the construct level. These numbers imply 36 percent shared variance between WCST categories and FSIQ at the construct level in comparison to 19 percent that shared variance at the measurement level (the Appendix A provides more details for disattenuation analyses). While these numbers indicate that the proportion of shared variance at the construct level might be about two times higher than the proportion of shared variance at the measurement level, they also indicate that—at best—about one third of the variability in mental shifting (i.e., the presumed construct behind performance on the WCST) can be accounted for by variability in the common construct of intelligence. The results from these disattenuation analyses hence suggest that the mental shifting construct is not identical to the commonly presumed intelligence construct. We conclude that WCST scores and IQ domains represent partially independent constructs, even if the attenuation of the correlations between them is taken into account. These results support the idea that, despite conceptual overlap between accepted definitions of intelligence and executive functioning, the WCST assesses cognitive abilities that are partially separable from intelligence. 

One of the reviewers asked for a report of internal reliability indices of a WCST version since our literature review concerning this matter was fruitless, as discussed above (the literature reports only indices of test-retest reliability). The question that arises here is whether the assumption of WCST internal reliability estimates of at least *r_yy_* = 0.60 is defendable because otherwise the construct-level correlation between WCST scores and IQ domains would surpass the calculated estimate of *r_xtyt_* = 0.60. Note, that we recently published internal reliability estimates of WCST scores, and that these estimates fell well above *r* = 0.90, suggesting that *r_yy_* = 0.60 yields a well-defendable estimate for the upper bound of construct-level correlations between WCST scores and IQ domains [9].

Hence, the WCST surpasses Cronbach’s hurdle of sufficient discriminant validity against common intelligence tests, thereby justifying the utilization of the mental shifting construct in the context of the WCST. Viewed from this broader perspective, the overlap between EF (or at least, a more specific EF shifting factor, represented by the WCST) and intelligence (represented by the most popular IQ tests) seems to corroborate earlier conclusions that were drawn from individual differences studies [31,35].

### 4.2. Differential Associations between WCST Scores and IQ Domains

The correlations between distinct WCST scores and the FSIQ showed some differences that may be worth a short comment. The average correlation between WCST categories and FSIQ was highest (*r* = 0.44), followed by the correlations between WCST total errors (*r* = −0.42), WCST perseverations (*r* = −0.39), WCST NPE (*r* = −0.29) and FSIQ, with the lowest correlations (close to zero) between WCST FMS and FSIQ (*r* = −0.05). Hence, differential correlations between WCST categories, total errors, perseverations and FSIQ appear negligible. Comparable correlations with FSIQ suggested that these WCST scores reflected, at least to some degree, a common cognitive ability (e.g., shifting, general EF, or general understanding of the task). This common cognitive ability seemed to be less relevant for the other two WCST scores that we analyzed (i.e., NPE and FMS), as suggested by lower correlations with FSIQ. Given the interdependencies of WCST scores, it seems plausible that a deficit which relates to an increase in the number of committed perseverations also increases total errors and decreases WCST categories, while leaving NPE and FMS more or less unaffected. 

Alternatively, the differential correlations between two groups of WCST scores (categories, total errors, perseverations vs. NPE, FMS) and FSIQ might be related to differences in their psychometric properties. Specifically, the first group of WCST scores may on average provide more reliable estimates in comparison with the second group (see Appendix A). In this case, the attenuation effect that was discussed above in detail would more strongly affect the second group of WCST scores than the first group. Hence, it is possible that all analyzed WCST measures are indicators of the ability that causes WCST performance to correlate with FSIQ, but that these indicators simply differ in how reliably they assess this ability. 

The issue of differential associations between WCST scores and IQ remains to be substantiated by future studies. This research should not only be concerned with differential associations between specific WCST scores and intelligence, but it should also be concerned with their reliability. As we have seen, inferences about validity hinge upon knowledge about the reliability that can be assumed for the scores under consideration. 

The correlations between WCST scores and distinct IQ domains followed the pattern *r*_WCST-FSIQ_ > *r*_WCST-VIQ_ = *r*_WCST-PIQ_ (see Table 2). The FSIQ advantage over verbal and non-verbal domains was of little surprise, given that the FSIQ yielded more reliable estimates (with an average split-half reliability across age groups of 0.98; cited following [13]) compared to the VIQ (with an average split-half reliability across age groups of 0.97 [13]), and compared to the PIQ (with an average split-half reliability across age groups of 0.94 [13]). Given the fact that the FSIQ comprised both, verbal and non-verbal domains, this difference in reliability was probably due to the positive effects of additional items on scale reliability [139]. The attenuation effect therefore exerted a slightly stronger influence on the WCST-VIQ and WCST-PIQ correlations compared to the WCST-FSIQ correlation.

WCST-VIQ and WCST-PIQ correlations were almost identical, perhaps with a marginal advantage for the verbal compared to the non-verbal domains. This finding may be interpreted as indicating that the predictability of WCST variance based on verbal domains, which are more closely related to Gc, does not differ from the predictability of WCST variance based on non-verbal domains, which are more closely related to Gf. The irrelevance of the contents of the particular IQ domains on the WCST-IQ correlations seems to be at odds with the common assumption that EF in general, and WCST scores in particular, are preferentially correlated with Gf, while the correlation with Gc is negligible [43,72,140]. However, reliability coefficients were slightly higher for the verbal domains compared to the non-verbal ones (see above; also, estimates of the NART test-retest reliability exceeded 0.95 [60], whereas reliability estimates for the RPM and CFT seemed to lie in the interval between 0.80 and 0.90; see [62] (cited after [141]) and [13], but see [142] for RPM test-retest reliability estimates > 0.90). Given the available reliability estimates, the predictability of WCST variance from verbal IQ domains may be actually lower than the predictability of WCST variance from non-verbal IQ domains, but this difference may be counteracted by differential degrees of attenuation due to slight differences in verbal and non-verbal IQ reliabilities. 

We suggest conducting specifically designed studies for a better understanding of the relationship between WCST scores and tests of crystallized and fluid intelligence. Notice that the conductance of such a study would be of theoretical importance since the enigma of unimpaired IQ in patients with frontal lobe damage and WCST deficits [15] was explained by shortcomings in the utilized intelligence tests, and specifically, insufficient measurement of fluid intelligence at that time [140]. Other researchers emphasized the association between the frontal lobes and fluid intelligence [43,72,143]. Tranel et al. [140], however, did not find evidence for an association between the presence of frontal lesions and decrements in the Matrix Reasoning subtest of the WAIS-III, rendering the issue controversial to what degree Gf mediated the association between frontal lesions and EF. Later work from this group [144,145] and other groups [43] pointed to the direction that general intellectual abilities draw on a circumscribed, albeit distributed, network in frontal and parietal lobes. A deeper review of the neural mechanisms of intelligence would go beyond the scope of this article, and the interested reader is referred to the relevant literature [43,146,147,148].

### 4.3. The Role of Moderator Variables

We also found considerable effect-size heterogeneity, and our moderator analyses could only account for small portions of this heterogeneity. Despite the fact that substantial portions of the study-by-study variability remain unexplained, our moderator analyses led to the identification of two potentially interesting moderators (i.e., age, clinical status). First, the correlations between WCST perseverations and non-verbal IQ were markedly stronger in samples with a mean age above 50 years than they were in samples of younger adults (18–50 years) or in samples of children and adolescents. Second, correlations between WCST perseverations and the FSIQ were larger in patient samples than in control samples. One account of these moderator effects holds generalized cognitive decline responsible, which could occur in some of the studied individuals, regardless of whether one looks at samples of healthy elderly people or at clinically relevant disease states. Generalized cognitive decline in some elderly or ill individuals should increase inter-individual variability on WCST scores and in IQ in these samples as compared to the usually more homogenous samples of young and healthy participants. To the extent that this cognitive decline is generalized, additional variance in WCST scores and in IQ domains would be shared, thereby increasing the correlation between them in elderly and ill samples. However, these moderator effects should be interpreted with caution, due to the low statistical power of the comparisons that we were able to carry out. Cautiousness is also suggested by the fact that these relationships did not occur consistently across all WCST-IQ combinations.

The number of data available for the analysis of partial, age-corrected WCST-IQ correlations was severely limited. Yet, there seemed to exist substantial WCST-IQ correlations even if the age of the participants was partialed out of the raw WCST-IQ correlations (*r* = 0.36). Note that these partial correlations might be larger for some WCST-IQ combinations. However, we had to pool the data across all available WCST scores and IQ domains, a procedure which implied that the partial correlation might have been attenuated through the inclusion of WCST scores that showed weaker correlations with IQ (i.e., WCST FMS).

### 4.4. Limitations of the Present Meta-Analysis and Suggestions for Future Studies

The opportunity to obtain more conclusive evidence from the present meta-analysis was restrained by a number of obstacles: First, most of the studies that we analyzed comprised relatively small sample sizes. The effect-size estimates that we could provide were based on quite a variable *N*s, ranging from *N* = 260 (WCST FMS-VIQ) to *N* = 3256 (WCST perseverations-PIQ). Although our bias analyses did not suggest that the size of the correlations covaried with the within-study standard errors (see Figure 2), the presence of these strongly unbalanced *N*s calls for more systematic studies. These studies should not only explore plausible associations (i.e., WCST perseverations-Gf, WCST perseverations-PIQ), but also less plausible associations (WCST FMS-Gc, WCST FMS-VIQ) with identical rigor in order to facilitate valid inferences about differential associations. 

Second, a lack of conceptual sharpness with regard to the structure of intelligence that was apparent in many studies prevented firm conclusions about differential associations of theoretical importance between WCST scores and crystallized and fluid intelligence, respectively. Neither EF, nor intelligence, can be considered as a unitary construct. In the case of EF, the available evidence points into the direction that WCST scores load on an isolable mental shifting factor [35,149]. Yet, it remains a viable possibility that mental shifting abilities share substantial portions of variance with a hitherto not identified cognitive ability that also underlies performance on common intelligence tests. As an example, studies of ‘relational’ reasoning that manipulated the number of to-be-integrated relations revealed that frontal lobes are critical for the integration of multiple relations [150,151,152,153,154] Successful WCST performance requires a comparable integration of information from multiple dimensions. Future studies should be more specifically designed toward theory-driven research questions, and they should primarily be concerned with isolating more circumscribed facets of WCST performance and intelligence alike.

Third, the role of reliability in understanding construct validity seems to be underrated. In fact, Schmidt and Hunter’s methods of meta-analysis [155] could not be applied due to the currently less than satisfactory knowledge about reliabilities of the WCST scores. No information about internal reliability was available in any of the published studies, and the data about test-retest reliability are by and large discouraging [9]. The apparently low reliability of WCST scores is certainly below levels considered acceptable in clinical practice (but see [9]), supporting the idea to merge them to a global index of executive functioning (as in Schretlen’s modified WCST version [39]). Further research should try to circumvent the reliability limitations of the WCST scores by appropriate efforts at improving this useful assessment technique. Future studies of WCST-IQ associations should preferably be conducted using psychometrically matched [156,157] measures of crystallized and fluid intelligence.

Taken together, these considerations imply the necessity to conduct large-scale, theory-driven, and psychometrically sound studies of EF-IQ associations in both healthy and clinical samples. We consider these studies of importance because construct validity increasingly gains importance in the field [74,158,159,160,161]. This is particularly true with regard to the discriminant validity of neuropsychological tests that are assumed to assess EF as a construct separable from standard conceptualizations of intelligence. The demand for construct validation, in particular the assurance of discriminant validity, is due to the progressively disregarded role of criterion validity, where evidence for a frontal localization of dysexecutive symptoms serves as a major criterion for test validity.

## 5. Conclusions

The analyzed data revealed low to medium-sized associations between WCST scores and IQ, which were not simply mediated by effects of age, suggesting that the two types of assessment shared portions of variance to a modest degree. The major finding of our meta-analysis was that, at best, about one third of the variability in the EF facet measured by the WCST (i.e., mental shifting [35,149]) could be accounted for by variability in common indicators of intelligence.

Our conclusion is that, despite conceptual overlap between accepted definitions of intelligence and executive functioning, the WCST assesses cognitive abilities that are partially separable from intelligence. Furthermore, our findings provide little evidence, if at all, for differential associations between distinct WCST scores and IQ. Although some of them (categories, total errors, perseverations) showed somewhat stronger associations with IQ than others (non-perseverative errors, failures to maintain set), it remains to be seen whether this differential association should be attributed to their contents, or alternatively, to their psychometric properties. WCST scores were associated in comparable strength with verbal and non-verbal IQ domains, thus not supporting the idea that the former were preferably associated with non-verbal/fluid intelligence. Overall, our meta-analysis represents a step toward evidence-based neuropsychology [162,163] by shedding light on the hitherto understudied discriminant validity of a widely used test of executive functions. 

## Figures and Tables

**Table 1:** Overview of the studies included in the meta-analysis.

First Author	Year	*N*	Sample	% Fem	Age (*M*)	Age (*SD*)	WCST Version	FSIQ	VIQ	PIQ	Cor
Ardila [84]	2000	50	children (healthy)	0.0	14.4	1.0	Heaton, 1981	WISC-R	WISC-R	WISC-R	
Bird [85]	2004	90	adults (healthy)	62.2	57.0	8.3	Nelson, 1976		NART		mix
Boone [86]	1998	250	adults (healthy and patients, psy & neuro)	46.0	55.5	15.5	Heaton, 1981		WAIS-R	WAIS-R	
Chien [87]	2009	99	adults (healthy)	0.0	20.2	0.6	Heaton, 1993 (c)	WAIS-R			
Cianchetti [88]	2007	101	children (healthy)	52.5	4.0	0.0	Nelson, 1976			RPM	P
Cianchetti	2007	113	children (healthy)	50.4	5.0	0.0	Nelson, 1976			RPM	P
Cianchetti	2007	119	children (healthy)	47.1	6.0	0.0	Nelson, 1976			RPM	P
Cianchetti	2007	161	children (healthy)	52.8	7.0	0.0	Nelson, 1976			RPM	P
Cianchetti	2007	94	children (healthy)	52.1	8.0	0.0	Nelson, 1976			RPM	P
Cianchetti	2007	98	children (healthy)	50.0	9.0	0.0	Nelson, 1976			RPM	P
Cianchetti	2007	119	children (healthy)	50.4	10.0	0.0	Nelson, 1976			RPM	P
Cianchetti	2007	122	children (healthy)	48.4	11.0	0.0	Nelson, 1976			RPM	P
Cianchetti	2007	100	children (healthy)	50.0	12.0	0.0	Nelson, 1976			RPM	P
Cianchetti	2007	99	children (healthy)	48.5	13.0	0.0	Nelson, 1976			RPM	P
Crawford [89]	2000	123	adults (healthy)	61.0	39.4	13.4	Nelson, 1976	WAIS-R	WAIS-R		P
Crawford	1999	90	adults (healthy)	55.6	72.8	6.5	Nelson, 1976	WAIS-R (s)			P
Davis [90]	2000	62	adults (healthy)	51.6	20.3	1.5	Heaton et al., 1993		WAIS-III (s)	PMA (s)	P
de Zubicaray [91]	1998	36	adults (healthy)	66.7	70.1	5.6	Nelson, 1976	WAIS-R	WAIS-R	WAIS-R	S
Dieci [92]	1997	88	adults (healthy and patients, psy)	28.4	27.3	7.0	Heaton, 1981	WAIS-R	WAIS-R	WAIS-R	S
Dolan [93]	2002	60	adults (patients, psy)	0.0	29.8	6.6	Heaton, 1981		NART		S
Evans [94]	2016	192	children (healthy)	52.1	12.4	1.8	Heaton et al., 1993	WASI (s)			P
Giovagnoli [95]	2001	26	adults (patients, neuro)		36.8	10.9	Nelson, 1976			RPM	S
Giovagnoli	2001	21	adults (patients, neuro)		33.3	11.2	Nelson, 1976			RPM	S
Giovagnoli	2001	18	adults (patients, neuro)		36.6	13.4	Nelson, 1976			RPM	S
Giovagnoli	2001	15	adults (patients, neuro)		41.4	9.8	Nelson, 1976			RPM	S
Giovagnoli	2001	14	adults (patients, neuro)		30.7	8.8	Nelson, 1976			RPM	S
Giovagnoli	2001	18	adults (patients, neuro)		32.6	12.2	Nelson, 1976			RPM	S
Giovagnoli	2001	30	adults (patients, neuro)		35.2	14.3	Nelson, 1976			RPM	S
Giovagnoli	2001	23	adults (patients, neuro)		35.6	13.4	Nelson, 1976			RPM	S
Giovagnoli	2001	36	adults (healthy)		36.1	10.7	Nelson, 1976			RPM	S
Golden [96]	1998	112	adults (patients, neuro)	48.2	37.4	13.3	Heaton, 1981	WAIS-R	WAIS-R	WAIS-R	P
Han [97]	2016	180	adolescents (healthy and patients, psy)	49.5	13.7	1.5	Heaton, 1981 (c)	K-BIT (s)			
Heinrichs [98]	1990	56	adults (patients, neuro)	30.4	43.8	13.6	Heaton, 1981	WAIS-R			P
Ilonen [99]	2000	27	adults (patients, psy)	63.0	33.0	13.6	Heaton et al., 1993	WAIS-R			S
Isingrini [100]	1997	35	adults (healthy)	57.1	35.5	7.6	Nelson, 1976		WAIS (s)	CM	
Isingrini	1997	72	adults (healthy)	48.6	80.6	8.6	Nelson, 1976		WAIS (s)	CM	
Keefe [101]	1994	54	adults (healthy and patients, psy)	59.3	34.8	10.5	Heaton, 1981		WAIS-R (s,v)	WAIS-R (s)	P
Kilincaslan [102]	2010	39	children (healthy and patients, psy)	15.4	12.2	2.7	Heaton, 1993 (c)	WISC-R	WISC-R	WISC-R	
Lee [103]	2009	39	adults (patients, psy)	51.3	32.4	7.2	Heaton, 1993 (c)	WAIS-R (s)			P
Lee	2009	33	adults (healthy)	57.6	29.0	8.9	Heaton, 1993 (c)	WAIS-R (s)			P
Lehto [104]	2003	51	children (healthy)	41.2	9.2	0.3	Heaton, 1981			RPM	P
Lehto	2003	40	adults (healthy)	62.5	30.1	9.6	Heaton, 1981			RPM	P
Lehto [105]	2004	46	children (healthy)	43.5	12.5	0.3	Heaton, 1981			RPM	P
LeMonda [106]	2012	44	children (patients, psy)	22.7	8.1	1.0	Heaton et al., 1993			WISC-R (s)/SB4(s)	
Lichtenstein [107]	2018	226	adults and adolescents (healthy and patients, psy)	35.4	13.6	2.6	Heaton, et al. 1993	WISC-III & WISC-IV			P
Lineweaver [108]	1999	229	adults (healthy)	57.6	69.1	8.6	Nelson, 1976	WAIS-R (s)	WAIS-R (s)	WAIS-R (s)	S
Liss [109]	2001	21	children (patients, psy)	14.3	9.2	0.3	Heaton et al., 1993	n/a	n/a	n/a	P
Liss	2001	34	children (patients, psy)	29.4	9.1	0.1	Heaton et al., 1993	n/a	n/a	n/a	P
Lucey [110]	1997	38	adults (healthy and patients, psy)	47.4	38.0	11.5	Heaton, 1981		NART		P
Minshew [111]	2002	90	adults and adolescents (patients, psy)		21.4	9.7	Heaton et al., 1993	WAIS-R			
Minshew	2002	107	adults and adolescents (healthy)		21.2	9.8	Heaton et al., 1993	WAIS-R			
Mullane [112]	2007	30	children (healthy and patients, psy)	26.7	8.8	1.2	Heaton et al., 1993	WISC-III (s)			P
Nestor [113]	2015	81	adults (healthy)		40.8	9.1	Heaton, 1981	WAIS-III	WAIS-III	WAIS-III	P
Obonsawin [114]	1999	146	adults and adolescents (healthy)	47.3	40.3	14.0	Nelson, 1976	WAIS-R	WAIS-R	WAIS-R	K
Obonsawin [115]	2002	123	adults (healthy)	38.2	40.3	14.0	Nelson, 1976	WAIS-R			P
Owashi [116]	2009	27	adults (patients, psy)	55.6	41.5	10.1	Heaton, 1993 (c)	WAIS-R (s)			S
Perry [117]	1998	71	adults (patients, psy)	60.6	34.2	8.7	Heaton, 1981		WAIS-R (s,v)		P
Roca [118]	2012	31	adults (healthy and patients, neuro)		60.6	8.0	Nelson, 1976			RCPM	
Roca [72]	2010	74	adults (healthy and patients, neuro)		49.9	12.6	Nelson, 1976			CFT (s)	P
Rossell [119]	2003	78	adults (patients, psy)	0.0	33.7	8.5	Heaton, 1981		NART		P
Salthouse [120]	1996	259	adults (healthy)	63.3	51.4	18.4	Heaton et al., 1993			WAIS-R (s)/SA	P
Schiebener [121]	2015	112	children (healthy)	52.7	13.6	3.4	Nelson, 1976 (c)			RPM	P
Shura [122]	2016	205	adults (patients, psy & neuro)	10.8	34.9	9.1	Heaton, 1981 (c)		WAIS-III (s)		P
South [123]	2007	19	children (patients, psy)	26.3	14.9	2.7	Grant, 1948	n/a	n/a	n/a	P
South	2007	18	children (healthy)	38.9	14.1	2.9	Grant, 1948	n/a	n/a	n/a	P
Steingass [124]	1994	101	adults (patients, psy)	21.9	50.5	8.1	Nelson, 1976	WAIS (s)	WAIS (s)/MWT-B (v)	WAIS (s)	P
Sweeney [125]	1991	44	adults (patients, psy)	40.9	28.5	8.6	Heaton, 1981		AQT (v)		
Syngelaki [126]	2009	70	adults and adolescents (healthy and patients, psy)	0.0	16.3	1.5	Heaton, 2005 (c)	WASI (s)			P
Taconnat [127]	2007	81	adults (healthy)	51.9	66.0	8.2	Heaton et al, 1993			CFT	P
Whiteside [128]	2016	304	adults (patients, neuro)	54.9	45.1	13.4	Heaton et al., 1993		WAIS-III (s,v)		P
Yasuda [129]	2014	33	adults and adolescents (patients, psy)	39.4	26.1	11.5	Kashima et al., 1987 (c)	WAIS-III	WAIS-III	WAIS-III	P
Yasuda	2014	33	adults and adolescents (healthy)	39.4	26.8	9.6	Kashima et al., 1987 (c)	WAIS-III	WAIS-III	WAIS-III	P

Note: % fem = percent female participants in the sample, age (M) = mean age in the sample, age (SD) = standard deviation of participants’ age, WCST = Wisconsin Card Sorting Test, FSIQ = full scale IQ, VIQ = verbal IQ, PIQ = performance IQ, cor = correlation coefficient, psy = psychiatric, neuro = neurological, (c) = computerized, (s) = short form/subscales, (v) = vocabulary test, WISC = Wechsler Intelligence Scale for Children, WAIS = Wechsler Adult Intelligence Scale, WASI = Wechsler Abbreviated Scale of Intelligence, R(C)PM = Raven’s (Colored) Progressive Matrices, NART = National Adult Reading Test, PMA = Primary Mental Abilities, K-BIT = Kaufman Brief Intelligence Test, CM = Cattell’s Matrices, SB4 = Stanford–Binet Intelligence Scale, CFT = Cattell Culture Fair Intelligence Test, AQT = Ammons Quick Test, Shipley Abstraction Test, MWT-B = Multiple Choice Word Test-B, P = Pearson’s *r*, S = Spearman’s *rho*, K = Kendall’s *tau*, mix = parametric and non-parametric correlations.

**Table 2:** Results of the meta-analyses of correlations between WCST performance and intelligence.

IQ Domain	Statistic	Categories	Perseverations	NPE	FMS	TE
	Number of samples (*k*)	20	25	6	6	11
	Significant correlations (%)	70	76	50	0	64
FSIQ	Total *N*	1533	2049	664	553	710
	Average effect size *r*	0.44	−0.39	−0.29	−0.05	−0.42
	[95% CI]	[0.36, 0.51]	[−0.45, −0.33]	[−0.46, −0.11]	[−0.14, 0.03]	[−0.51, −0.31]
	*Q*	63.31 *	50.41 *	26.56 *	1.34	22.02 *
	*I*²	68.41	48.42	73.64	0	45.50
	τ_Begg & Mazumar_	−0.08	−0.26	0.07	−0.07	0.15
	*p*_Begg & Mazumar_	0.626	0.076	0.851	0.851	0.529
	Number of samples (*k*)	19	24	6	4	11
	Significant effects (%)	74	71	67	0	64
VIQ	Total *N*	1755	2071	546	260	871
	Average effect size *r*	0.33	−0.31	−0.30	−0.02	−0.37
	[95% CI]	[0.26, 0.39]	[−0.36, −0.26]	[−0.44, −0.16]	[−0.15, 0.10]	[−0.45, −0.29]
	*Q*	37.08 *	30.99	14.33 *	3.03	17.01
	*I*²	46.06	19.33	51.15	0	29.45
	τ_Begg & Mazumar_	−0.20	−0.01	−0.20	1	0.18
	*p*_Begg & Mazumar_	0.234	0.941	0.573	−	0.435
	Number of samples (*k*)	28	42	17	14	22
	Significant effects (%)	75	52	53	14	73
PIQ	Total *N*	2506	3256	1784	1386	2015
	Average effect size *r*	0.34	−0.29	−0.19	−0.08	−0.36
	[95% CI]	[0.27, 0.39]	[−0.34, −0.24]	[−0.27, −0.11]	[−0.13, −0.02]	[−0.42, −0.29]
	*Q*	69.75 *	88.87 *	44.76 *	14.70	62.07 *
	*I*²	58.42	51.61	59.79	0	62.95
	τ_Begg & Mazumar_	0.05	0.17	0.14	−0.11	−0.11
	*p*_Begg & Mazumar_	0.693	0.121	0.433	0.584	0.498

Note: Significant correlations (%) = percentage of included correlation coefficients with a 95% confidence interval excluding 0. * significant heterogeneity at α = 0.05. WCST = Wisconsin Card Sorting Test, NPE = non-perseverative errors, FMS = failures to maintain set, TE = total errors, FSIQ = full scale IQ, VIQ = verbal IQ, PIQ = performance IQ.

**Table 3:** Results of the meta-regression analyses conducted to examine the role of potential moderators of WCST-intelligence relationships.

Moderator	Categories					Perseverations				
Continuous moderators	β	95% CI	df	*t*	*p*	β	95% CI	df	*t*	*p*
FSIQ										
Mean age	0.00	[−0.09, 0.11]	17	0.15	0.880	0.00	[−0.08, 0.07]	22	−0.21	0.837
SD age	0.07	[−0.05, 0.18]	17	1.15	0.268	−0.04	[−0.11, 0.04]	22	−0.93	0.362
Percent female	0.01	[−0.09, 0.12]	14	0.27	0.795	0.01	[−0.06, 0.09]	19	0.35	0.733
VIQ										
Mean age	−0.02	[−0.09, 0.06]	16	−0.49	0.633	0.00	[−0.06, 0.06]	21	0.64	0.949
SD age	0.06	[−0.03, 0.15]	16	1.35	0.195	−0.03	[−0.10, 0.04]	21	−0.73	0.471
Percent female	0.01	[−0.06, 0.08]	15	0.34	0.742	0.05	[−0.04, 0.07]	20	0.57	0.573
PIQ										
Mean age	0.04	[−0.01, 0.10]	25	1.60	0.122	−0.07 *	[−0.11, −0.02]	39	−3.05	0.004
SD age	0.04	[−0.02, 0.11]	25	1.33	0.197	−0.03	[−0.08, 0.01]	39	−1.38	0.174
Percent female	0.00	[−0.09, 0.10]	23	0.06	0.951	−0.01	[−0.10, 0.08]	29	−0.25	0.801
Categorical moderators	χ^2^		df		*p*	χ^2^		df		*p*
FSIQ										
Age group	1.14		2		0.566	2.89		2		0.236
Clinical status	2.60		1		0.107	4.76 *		1		0.029
WCST version	0.01		1		0.911	1.16		1		0.282
WCST administration	2.71		1		0.100	2.33		1		0.127
IQ test type	0.33		1		0.567	2.93		1		0.087
VIQ										
Age group	1.81		2		0.405	0.48		2		0.787
Clinical status	0.26		1		0.609	0.00		1		0.972
WCST version	0.30		1		0.586	1.37		1		0.242
WCST administration	2.78		1		0.095	0.83		1		0.363
IQ test type	0.25		1		0.617	0.02		1		0.888
PIQ										
Age group	2.893		2		0.235	13.77 *		2		0.001
Clinical status	3.431		1		0.064	0.18		1		0.669
WCST version	0.41		1		0.522	3.78		1		0.052
Categorical moderators	χ^2^		df		*p*	χ^2^		df		*p*
PIQ WCST administration	0.42		1		0.518	0.06		1		0.810
IQ test type	0.03		1		0.858	2.55		1		0.110

Note: * significant moderator at α = 0.05. WCST = Wisconsin Card Sorting Test, FSIQ = full scale IQ, VIQ = verbal IQ, PIQ = performance IQ.

## Appendix A

The available reliability studies of common WCST measures are summarized in Table A1. It is worth noting that our compilation of reliability studies does not pretend being exhaustive because our search for suitable studies was not systematic. Inspection of Table A1 reveals that estimates of WCST internal consistency remained completely unavailable.

All studies relied on repeated administration of the WCST, estimating test-retest-reliabilities with variable retest periods. Nine studies examined the administration of a WCST version in non-clinical populations (cumulative *N* = 568), implying that the five studies of clinical populations achieved a cumulative *N* of only 120 patients. In addition, the diagnoses under consideration were quite heterogeneous (traumatic brain injury, sleep apnoe syndrome, autism, learning disability). Very little is known about test-retest reliability when the WCST is administered in clinical populations that are of major interest for neuropsychologists. In that regard, one has to rely on the estimates from those two studies that looked at patients who suffered from traumatic brain injury (cumulative *N* = 57 [164,165]. However, the results from these two studies can hardly be considered as being convergent.

Many different coefficients were considered across these studies (Pearson’s *r*, Spearman’s *rho*, Kendall’s *tau*, intra-class correlations, generalizability coefficients). Most of the studies were based on quite small sample sizes. Charter [166] showed that precise reliability estimates presuppose sample sizes of *N* > 400. In order to obtain reliable estimates of WCST test-retest reliabilities, we averaged across all available studies, thereby ignoring the methodological differences between them, including the population under study, the duration of the retest period, the WCST version, and the type of coefficient under consideration. Given that reliability is the result of a test administered under particular circumstances to the sample under study, we also report averaged WCST test-retest reliability coefficients separately for samples (non-clinical, clinical) and test versions. The results are shown in Table A2.

**Table A1:** A summary of published studies on the WCST reliability.

Study	Year	Population	*N*	Type of Reliability	Test-Retest Interval	WCST Version	WCST Measure	Coefficient	Type of Correlation
Basso et al. [167]	1999	Non-clinical	50	Test-retest	12 months	Heaton et al. 1993	CAT TE PE, P FMS	0.54 0.50 0.52, 0.50 −0.02	6 6 6 6
Bird et al. [85]	2004	Non-clinical	90	Test-retest	1 month	Nelson 1976	TE PE	0.34 0.38	1 1
Bowden et al. [168]	1998	Non-clinical	75	Test-retest(‘alternate’ forms)	Same day	Heaton et al. 1981	CAT TE PE, P NPE	0.60 0.51 0.32, 0.30 0.43	2 2 2 2
de Zubicaray et al. [91]	1998	Non-clinical	36	Test-retest	7.5 months	Nelson 1976	CAT TE PE NPE FMS	0.28 0.36 0.27 0.38 0.49	2 2 2 2 2
Greve et al. [164]	2002	TBI	34	Test-retest	66 weeks	Heaton et al. 1993	CAT TE PE, P NPE FMS	0.53 0.82 0.80, 0.78 0.50 0.26	3 2 2 2 3
Heaton et al. [38]	1993	Non-clinical	46	Test-retest	33 days	Heaton et al. 1993	TE PE, P NPE	0.71 0.52, 0.53 0.72	5 5 5
Ingram et al. [169]	1998	Sleep apnoe patients	29	Test-retest	12 days	Computerized WCST	CAT TE PE, P FMS	0.70 0.79 0.83, 0.79 0.50	2 2 2 2
Lineweaver et al. [108]	1999	Non-clinical	142	Test-retest	24 months	Nelson 1976	CAT PE NPE	0.56 0.64 0.46	1 1 1
Ozonoff [169]	1995	Autistic children	17	Test-retest	30 months	Standard WCST	TE P	0.94 0.93	5 5
		Learning disabled children and adolescents	17	Test- retest	30 months	Standard WCST	TE P	0.90 0.94	5 5
Paolo et al. [170]	1996	Non-clinical	87	Test-retest	12 months	Heaton et al. 1981	CAT TE PE, P NPE FMS	0.65 0.66 0.65, 0.63 0.55 0.13	3 3 3 3
Steinmetz et al. [171]	2010	Non-clinical	22	Test-retest	Same day	Heaton et al. 1993	TE PE FMS	0.68 0.72 0.16	6 6 6
Tate et al. [165]	1998	Non-clinical	20	Test-retest	8 months	Heaton et al. 1993	CAT TE PE, P NPE FMS	0.88 0.79 0.72, 0.68 0.74 −0.04	2 4 4 4 4
		TBI	23	Test-retest	10 months	Heaton et al. 1993	CAT TE PE, P	0.29 0.39 0.34, 0.33	2 4 4
							NPE FMS	0.32 −0.32	4 4

*Note.* CAT = categories; TE = total errors; PE, P = perseveration errors, perseverations; NPE = non-perseverative errors; FMS = failures to maintain set; TBI = traumatic brain injury; 1 = Pearson’s *r*, 2 = Spearman’s *rho*, 3 = Kendall’s *tau*, 4 = intra-class coefficient, 5 = generalizability coefficient, 6 = unspecified; * Study-wise *N* served as the weighting factor.

Several observations deserve comments: First, average weighted test-retest reliabilities of WCST number of categories, total errors, and perseverations appeared to be very similar, and these coefficients achieved average values close to 0.60. Non-perseverative errors seem to show slightly lower average test-retest reliabilities (about 0.50). Failures to maintain set achieved a very low average test-retest reliability of about 0.15.

**Table A2:** Summary statistics of WCST reliability estimates.

**Overall and Sample-Specific**	**WCST Measure**	**Cumulative *N***	**Unweighted Average**	**Weighted Average ***
all (14) available samples	CAT TE PE, P NPE FMS	496 546 688 463 301	0.56 0.65 0.61 0.51 0.15	0.55 0.58 0.56 0.50 0.16
non-clinical (9) samples	CAT TE PE, P NPE FMS	410 426 568 406 215	0.59 0.57 0.52 0.55 0.14	0.58 0.53 0.52 0.51 0.14
clinical (5) samples	CAT TE PE, P NPE FMS	86 120 120 57 86	0.51 0.77 0.76 0.41 0.15	0.52 0.76 0.77 0.43 0.19
**Test Versions (excl. cWCST)**	**WCST Measure**	**Cumulative *N***	**Unweighted Average**	**Weighted Average ***
Heaton et al. version (10) samples	CAT TE PE, P NPE FMS	345 391 391 285 236	0.58 0.69 0.64 0.54 0.03	0.59 0.65 0.57 0.53 0.06
Nelson version (3) samples	CAT TE PE, P NPE FMS	178 126 268 178 36	0.42 0.35 0.43 0.42 0.49	0.50 0.35 0.50 0.44 0.49

*Note.* cWCST = computerized WCST; CAT = categories; TE = total errors; PE, P = perseverations; NPE = non-perseverative errors; FMS = failures to maintain set; *Study-wise *N* served as the weighting factor.

Second, reliability estimates appeared to be higher in clinical (0.7 < *r* < 0.8) in comparison to non-clinical (0.5 < *r* < 0.55) samples on two WCST measures (i.e., ‘number of total errors’, ‘perseverations’). The apparently low reliability of WCST measures often brings the argument forward that the WCST should not be utilized in clinical practice. However, these numbers suggest that appropriate studies should be conducted to reappraise the relatively good reliability of WCST perseverations in clinical samples because this measure represents the theoretically and clinically most relevant WCST measure.

Third, there was a general tendency toward lower test-retest reliability coefficients when retest periods were short: The Pearson correlation between the duration of that period (in days) and the reported coefficient for WCST perseverations amounted to *r* = 0.57 (*p* < 0.05 with *N* = 14 duration-coefficient pairings), indicating that procedural learning during the first test administration affects task performance on the second test administration over brief retest periods. This finding supports the idea that the WCST is a one-shot test that can only be administered once with any particular patient over brief time periods [13].

Fourth, with the remarkable exception of ‘failures to maintain set’, the Heaton and colleagues’ WCST versions [37,38] achieved slightly higher test-retest reliability coefficients compared to the Nelson version [36]. This putative version effect, if true, should probably be attributed to test length [139] because Heaton and colleagues’ WCST versions utilize 64 or 128 trials, whereas Nelson’s version is based on 48 trials.

Table A3 shows construct-level WCST-IQ correlations for a set of observed IQ-WCST correlations (0.05 < *r_xy_* < 0.50, reflecting the range of observed coefficients that we found in our meta-analysis). Construct-level WCST-IQ correlations result from the application of Spearman’s attenuation formula [137]

(A1) $$r_{x_{t}y_{t}}=\frac{r_{xy}}{\sqrt{r_{xx}r_{yy}}}r_{x_{t}y_{t}}=\frac{r_{xy}}{\sqrt{r_{xx}r_{yy}}}$$

with $r_{x_{t}y_{t}}$ representing a construct-level correlation, $r_{xy}$ representing an observed, measurement-level correlation, and $r_{xx}$ and $r_{yy}$ representing reliabilities of variables *x* (IQ; 0.90 < *r_xx_* < 0.99) and *y* (WCST; 0.10 < *r_yy_* < 0.60), respectively.

**Table A3:** Construct-level correlations, for different values of observed correlations (0.05, 0.1, 0.15, …, 0.5), IQ reliability (0.90, 0.95, 0.99), and WCST reliability (0.1, 0.2, 0.3, 0.4, 0.5, 0.6).

Observed Correlation	IQ Reliability	WCST Reliability					
		0.10	0.20	0.30	0.40	0.50	0.60
**0.05**	**0.90**	0.167	0.118	0.096	0.083	0.075	0.068
	**0.95**	0.162	0.115	0.094	0.081	0.073	0.066
	**0.99**	0.159	0.112	0.092	0.079	0.071	0.065
**0.10**	**0.90**	0.333	0.236	0.192	0.167	0.149	0.136
	**0.95**	0.324	0.229	0.187	0.162	0.145	0.132
	**0.99**	0.318	0.225	0.183	0.159	0.142	0.130
**0.15**	**0.90**	0.500	0.354	0.289	0.250	0.224	0.204
	**0.95**	0.487	0.344	0.281	0.243	0.218	0.199
	**0.99**	0.477	0.337	0.275	0.238	0.213	0.195
**0.20**	**0.90**	0.667	0.471	0.385	0.333	0.298	0.272
	**0.95**	0.649	0.459	0.375	0.324	0.290	0.265
	**0.99**	0.636	0.449	0.367	0.318	0.284	0.259
**0.25**	**0.90**	0.833	0.589	0.481	0.417	0.373	0.340
	**0.95**	0.811	0.574	0.468	0.406	0.363	0.331
	**0.99**	0.795	0.562	0.459	0.397	0.355	0.324
**0.30**	**0.90**	1.00	0.707	0.577	0.500	0.447	0.408
	**0.95**	0.973	0.688	0.562	0.487	0.435	0.397
	**0.99**	0.953	0.674	0.550	0.477	0.426	0.389
**0.35**	**0.90**	1.00	0.825	0.674	0.583	0.522	0.476
	**0.95**	1.00	0.803	0.656	0.568	0.508	0.464
	**0.99**	1.00	0.787	0.642	0.556	0.497	0.454
**0.40**	**0.90**	1.00	0.943	0.770	0.667	0.596	0.544
	**0.95**	1.00	0.918	0.749	0.649	0.580	0.530
	**0.99**	1.00	0.899	0.734	0.636	0.569	0.519
**0.45**	**0.90**	1.00	1.00	0.866	0.750	0.671	0.612
	**0.95**	1.00	1.00	0.843	0.730	0.653	0.596
	**0.99**	1.00	1.00	0.826	0.715	0.640	0.584
**0.50**	**0.90**	1.00	1.00	0.962	0.833	0.745	0.680
	**0.95**	1.00	1.00	0.937	0.811	0.725	0.662
	**0.99**	1.00	1.00	0.917	0.795	0.711	0.649

*Note.* IQ = intelligence quotient; WCST = Wisconsin Card Sorting Test.
